# Quantification of the immunohistochemical staining of fibroblast activation protein in intrathoracic solitary fibrous tumors using QuPath

**DOI:** 10.1007/s00595-025-03024-y

**Published:** 2025-03-24

**Authors:** Akiisa Omura, Toru Kimura, Tomohiro Maniwa, Tadashi Watabe, Keiichiro Honma, Yasushi Shintani, Jiro Okami

**Affiliations:** 1 Department of General Thoracic Surgery, Osaka International Cancer Institute, Osaka, Japan; 2 Department of General Thoracic Surgery, Osaka University Graduate School of Medicine, 2-2-L5, Yamadaoka, Suita, Osaka 565-0871 Japan; 3 Department of Nuclear Medicine and Tracer Kinetics, Osaka University Graduate School of Medicine, Suita, Japan; 4 Department of Diagnostic Pathology and Cytology, Osaka International Cancer Institute, Osaka, Japan

**Keywords:** Pleural tumor, Solitary fibrous tumor, Fibroblast activation protein, Malignant potential, Diagnostic target

## Abstract

**Purpose:**

Solitary fibrous tumors (SFTs) are rare mesenchymal neoplasms that can develop in the pleura. In the past, SFTs were considered benign, but there have been reports of SFTs being highly malignant. Fibroblast activation protein (FAP) is a serine protease, overexpressed in various cancers, which has been explored as a diagnostic and therapeutic target. We analyzed patients who underwent resection of an intrathoracic SFT, including metastatic pulmonary nodules from extrathoracic organs.

**Methods:**

The subjects of this retrospective study were seven patients with a primary SFT and two with metastatic SFTs in the lungs. After immunohistochemical staining of the resected tumors, quantification of the stained area was performed using QuPath.

**Results:**

Immunohistochemical quantification of FAP showed that it was expressed to varying degrees in the intrathoracic SFTs, with higher expression levels observed in metastatic SFTs than in primary pleural SFTs. Pathological examination confirmed the expression of FAP.

**Conclusion:**

Our results support the potential usefulness of FAP in the diagnosis of intrathoracic SFTs, including metastatic pulmonary nodules.

## Introduction

Solitary fibrous tumors (SFTs) are rare mesenchymal fibroblastic neoplasms that most often develop in the pleura [1]. Most SFTs grow slowly, but occasionally, they progress to an advanced malignancy. Malignant transformation can occur independently of the primary organ, with reports of extrathoracic SFTs spreading to pulmonary metastases [2, 3]. Fibroblast activation protein (FAP) is a serine protease upregulated in abnormally activated stromal fibroblasts in many epithelial cancers and sarcomas [4]. An increasing body of evidence suggests that [68 Ga] fibroblast activation protein inhibitor-46 (FAPI) positron emission tomography (PET) approaches are valuable diagnostic options [5]. Recently, FAP-targeted therapies have been investigated [6] and radionuclide therapies targeting FAP have been developed [7]. The antitumor effects of antibody–drug conjugates targeting FAP have been observed [8] and a phase I study of AVA6000, a peptide drug conjugate that releases doxorubicin in the tumor microenvironment of FAP-positive solid tumors, showed antitumor effects while reducing systemic exposure to doxorubicin [9]. As described, there has been significant development in the theranostics of FAP in recent years.

Recently, some case series on FAP expression in SFTs have been reported [10, 11]. However, there are no reports on the quantified expression levels of FAP in intrathoracic SFTs and pulmonary metastatic nodules originating from extrathoracic SFTs. In the present study, we quantitatively evaluated FAP expression in surgically resected intrathoracic SFT tumor tissues, as we hypothesized that intrathoracic SFTs, which include SFTs of the pleura and pulmonary metastatic nodules derived from other organs, could be considered as diagnostic targets.

## Methods

This retrospective study analyzed nine patients who underwent resection of a primary SFT of the pleura or pulmonary metastasis of an SFT. This study consisted of the main cohort of eight patients (nos. 1–8) and one patient who underwent FAPI PET imaging (no. 9). The main cohort included patients who underwent surgery at Osaka International Cancer Institute between 2006 and 2019. To guide the comparison between histological expression and FAPI PET imaging, we included one patient (no. 9) who underwent FAPI PET imaging at Osaka University Hospital: This case was reported previously by Kimura et al. [11]. In their report, FAPI PET was performed with an SUVmax of 3.57 in patient no. 9 [11]. In our study, age, sex, tumor size, and tumor location were included in the clinical evaluation. All medical information was obtained from medical records and the primary physician. Fluorine-18 fluorodeoxyglucose (18F-FDG) PET-CT was performed in four patients with a primary SFT of the pleura. This study was approved by the Institutional Review Board of the Osaka International Cancer Institute (#19110) and the Institutional Review Board of Osaka University Hospital (#21472–4). The requirement to obtain informed consent was waived.

We evaluated the FAP expression using immunohistochemical staining for all patients in this cohort. For immunohistochemical identification of FAP-positive cells after antigen retrieval, tissue slides were stained with rabbit anti-human FAP monoclonal IgG (1:245, 3 g/mL, clone EPR20021, #ab207178; Abcam). For the isotype control staining, the same concentration of rabbit IgG (#011-000-003; Jackson ImmunoResearch, West Grove, PA, USA) was used. For antigen retrieval corresponding to these antibodies, the slides were treated with 10 mM sodium citrate buffer containing 0.05% Tween for 20 min at 100 °C. The secondary antibody (goat anti-rabbit IgG H and L [HRP polymer], #ab214880; Abcam) was detected using 3,3’-diaminobenzidine (DAB). A whole-slide imaging analysis was performed using a slide imaging system (SlideView VS200; Olympus, Tokyo, Japan) to capture image data. FAP immunostaining of all samples was quantified using the open-source software program QuPath (ver. 0.5.1) with whole-slide data at 20 × magnification. Whole-slide images were analyzed using QuPath for image processing [12]. The H&DAB staining intensity of whole-slide images was optimized using the Estimated Staining Vector (ESV) feature of QuPath, as previously described [13]. FAP positivity was calculated as the percentage of DAB-positive areas relative to the total tissue area. The total tissue area was calculated with the thresholder function in QuPath using the following coefficients: extremely low resolution, average channel, Gaussian pre-filter, smoothing sigma 2, and threshold 230. The DAB-positive area was calculated using the following coefficients: high resolution, DAB channel, Gaussian pre-filter, smoothing sigma 1, and threshold of 0.1. A two-tailed Student’s *t*-test was conducted to evaluate any differences in FAP positivity between primary and metastatic SFTs. A scatter plot was created to visually represent the relationship between FAP positivity and the FAP H-score. A linear regression line was then fitted to the data, and Pearson's correlation coefficient was calculated to quantify the strength of the linear relationship. All statistical analyses were performed using the EZR software program (version 1.55).

As a supplementary study, we analyzed the correlation between our quantitative positivity and the diagnostic scores (FAP H-score) reported previously by Kimura et al. [11]. Therefore, we used the SFT case subjected to FAPI PET imaging as a guide for comparing the FAP intensity and SUV value of FAPI PET. The FAP positivity of all 13 patients listed in their article was quantified using the above method. A single regression analysis was performed for both scores to calculate correlation coefficients. Further information on the FAP H-score is described in reference [11].

## Results

Table 1 summarizes the data for all nine patients. Seven patients had a primary SFT and two had an SFT with pulmonary metastasis. Patient 7 had a primary tumor in the left knee and patient 8 had a primary tumor in the abdominal cavity. All patients had an SFT in the visceral pleura or lungs, except for patient no. 2, whose SFT had a parietal pleural origin. All the other patients underwent partial pulmonary resection of the lesion. For patient no. 2, the tumor was resected along with the parietal pleura. All patients with a primary SFT survived without recurrence until the last follow-up date. All patients with metastatic SFT died of the malignancy.

**Table 1:** Clinical data and immunostaining analysis

Patient no.	Age	Sex	Primary or metastatic	Location	Tumor size (mm)	FDG PET SUVmax	Mitoses /10HPF	FAP positivity (%)	Follow-up months)
1	57	M	Primary	Left lower lobe	78	2.7	3	9.34	NED (48)
2	53	M	Primary	Right anterior pleura wall	28	1.1	0	7.36	NED (1)
3	39	F	Primary	Right lower lobe	47	2.2	0	18.35	NED (12)
4	70	F	Primary	Left lower lobe	60	NA	0	5.66	NED (94)
5	73	F	Primary	Left upper lobe	52	1.6	0	12.31	NED (43)
6	63	F	Primary	Right upper lobe	36	NA	0	3.34	NED (1)
7	57	M	Metastatic	Right upper and lower lobe	15	NA	5	22.48	DOD (26)
8	48	M	Metastatic	Right upper and lower lobe	58	NA	3	86.37	DOD (19)
9	61	M	Primary	Right upper lobe	11	NA	0	12.31	NED (12)

PET-CT, positron emission tomography computed tomography; SUV, standardized uptake value; HPF, high power field; FAP, fibroblast activation protein; NA, not available; NED, no evidence of disease; DOD, died of disease

**Fig. 1:**
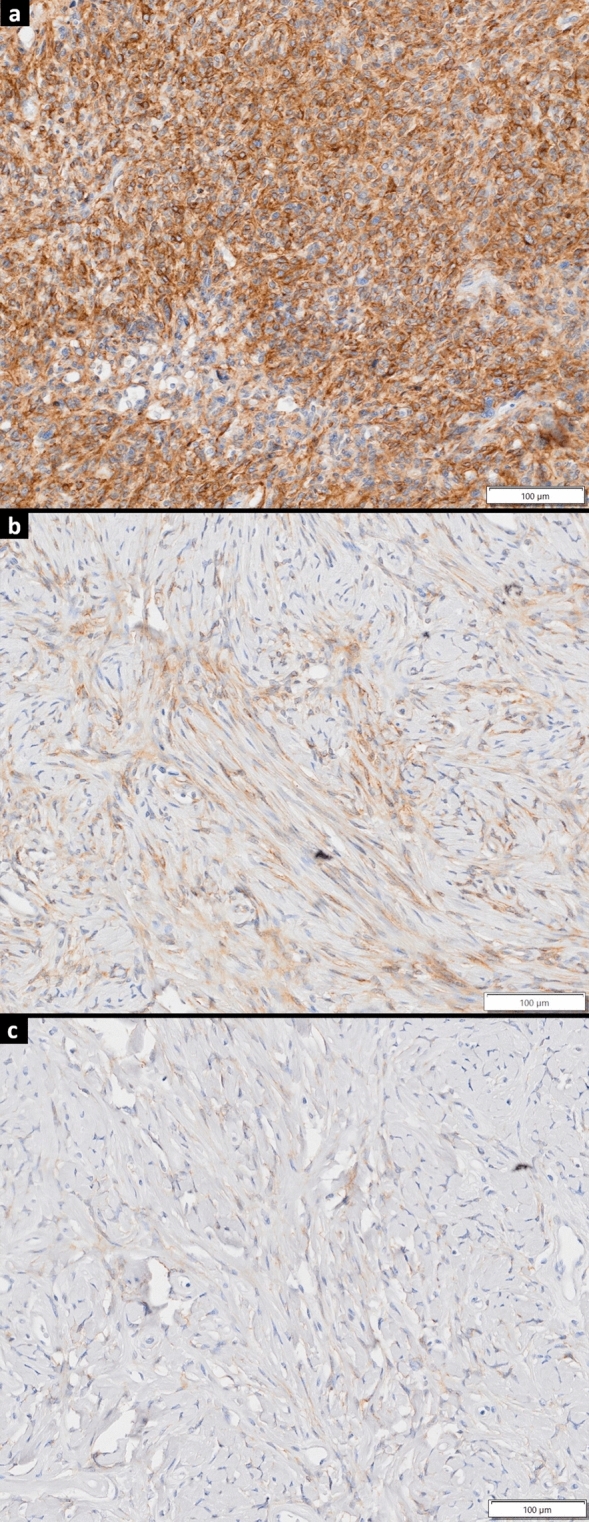
Immunohistostaining findings of fibroblast activation protein (FAP) in patients with a solitary fibrous tumor (SFT). Pathological findings are displayed at ×100 magnification. SFTs expressed varying degrees of FAP positivity. **a** Immunostaining findings in patient no. 8: staining was observed around spindle-shaped tumor cells. **b** Immunostaining findings in patient no. 3: sparse staining was observed around spindle-shaped tumor cells. **c** In this patient (no. 6), there was slight 3,3′-diaminobenzidine (DAB) staining

As shown in Table 1 and Fig. 1, the SFTs showed varying degrees of FAP positivity. The two patients with metastatic SFT (average FAP positivity, 54.4%) tended (*p* = 0.016) to have a higher degree of FAP positivity than the other patients (average FAP positivity 9.8%). Patient no. 9, who underwent FAPI PET with an SUVmax of 3.57, had a 12.31% FAP positivity. No significant correlation between FAP positivity and SUVmax was observed in the four patients with FDG PET results. The pathological outlines of all nine patients are shown in Online Resource 1. These images suggest that the FAP-positive area was located in the tumor nest rather than in the capsule surrounding the tumor nest.

As a supplementary study, FAP positivity, which was quantitatively evaluated using QuPath, was compared with the FAP H-score reported by Kimura et al. (Online Resource 2). The FAP positivity and the FAP H-score had a strong correlation (correlation coefficient 0.79, 95% confident interval 0.42–0.93, *p* = 0.0013), as shown in Online Resource 3.

## Discussion

This study found that intrathoracic SFTs, including SFTs of the pleura and metastatic pulmonary nodules of other organ-derived SFTs, expressed FAP by immunohistochemical staining. Moreover, we quantitatively evaluated FAP expression using QuPath and found that metastatic pulmonary nodules from an extrathoracic SFT tended to show higher FAP expression levels.

SFTs are rare mesenchymal spindle cell tumors that most commonly develop in the thoracic cavity, usually originating from the pleura [14, 15]. Therefore, a definitive pathological diagnosis without surgery is often difficult. It has been pointed out that ^18^F-FDG PET-CT findings have limited diagnostic ability for SFT and lack specificity [16]. Surgery is the usual treatment for an SFT [14], but recurrence and metastasis have been reported in a malignant subset of SFTs and are classified as malignant solitary fibrous tumors of the pleura (MSFTP) [15]. However, the extent of SFT resection is often the same as that of benign tumors, sometimes resulting in recurrence and reoperation owing to incomplete surgical margins [17]. Therefore, if a more precise preoperative diagnosis is available, it may be possible to plan surgery with a more secure resection margin during the initial surgery.

FAP is persistently expressed in various tumors. Recently, Crane et al. reported the expression of FAP in sarcomas, including a pathological report of an extrathoracic SFT [18]. Consistent with a previous pathological review of SFT, which reported that the histological appearance predominantly consists of a proliferation of tumor cells with abundant cytoplasm and minimal stroma [19], our findings of whole images suggest that stromal localization of FAP-positive cells is likely to be limited in SFTs.

The FAPI PET uptake and FAP immunostaining intensity have been suggested to be related to malignancy in pancreatic tumors [20]. Considering that pulmonary metastasis represents evidence of oncological aggressiveness, it seems logical that the FAP intensity of pulmonary metastatic nodules tended to be higher than that of other intrathoracic SFTs. Because FAP is overexpressed in tumors and tissue remodeling sites and its expression is difficult to detect in non-diseased adult organs, it has recently been reported as a tumor-specific therapeutic and diagnostic target [4]. Recently, FAP inhibitors have been developed as diagnostic tracers, and FAPI PET imaging has shown tumor-specific diagnostic performance in many cancers [21]. A report comparing FAPI PET with conventional FDG PET suggested the usefulness of FAPI PET [22]. Rasinski et al. also reported that FAPI PET is useful for assessing the malignancy of pancreatic cancer [23]. Their study showed an optimal SUV cutoff value that could discriminate malignancy with good sensitivity and specificity and suggested that FAPI PET can be applied clinically in the diagnosis of malignancy when imaging study findings are not definitive. FAPIPET has recently been shown to be diagnostically useful for lung cancer [24]. FAPI PET has also been reported to have better tumor affinity and a higher tumor-to-background ratio than other imaging methods [25]. Several recent reports have evaluated the tumor diagnostic potential of FAP immunostaining in intrathoracic SFTs [10, 11]. Our results, including a supplementary study, support their proposal regarding the oncologic importance of FAPI PET in patients with an SFT. In light of these studies, if differences in the histological expression of FAP are detectable by FAPI PET, it may also enable the diagnosis of intrathoracic SFT by the SUV value of FAPI PET. Furthermore, the FAP intensity of pulmonary metastatic nodules of extrathoracic SFT in this study suggests that FAPI PET may be able to distinguish between intrathoracic SFT as a primary lesion and pulmonary metastatic nodules of extrathoracic SFTs.

This study has several limitations. First, because it was a single-center study conducted only in Japan, it could not demonstrate a comparable pathological diagnosis at other institutions. Second, because it was a small case series report, we do not have sufficient evidence to suggest using FAP as an indicator of malignancy for SFTs. Third, this study does not provide oncogenic evidence of a causal relationship between the malignant transformation of SFTs and the hyperexpression of FAP. Finally, since the present study focused on FAP-targeted diagnoses rather than therapeutic applications for SFTs, no specific investigation was conducted to determine whether FAP expression is localized within tumor cells or the tumor stroma. Further case studies and large-scale investigations are necessary to address these limitations.

In conclusion, we confirmed that the tumor tissue of intrathoracic SFTs, including SFTs of the pleura and pulmonary metastatic nodules from other organ-derived SFTs, expressed FAP. The expression of FAP in intrathoracic SFTs, including metastatic pulmonary nodules of extrathoracic SFT, might be useful for imaging-based diagnoses and therapy.
